# Exploring Consumer Emotions in Pre-Pandemic and Pandemic Times. A Sentiment Analysis of Perceptions in the Fine-Dining Restaurant Industry in Bucharest, Romania

**DOI:** 10.3390/ijerph182413300

**Published:** 2021-12-17

**Authors:** Jacqueline-Nathalie Harba, Gabriela Tigu, Adriana AnaMaria Davidescu

**Affiliations:** 1 Doctoral School of Business Administration, Bucharest University of Economic Studies, 010374 Bucharest, Romania; jacqueline_harba@yahoo.com; 2 Department of Tourism and Geography, Bucharest University of Economic Studies, 010404 Bucharest, Romania; gabriela.tigu@ase.ro; 3 Department of Statistics and Econometrics, Bucharest University of Economic Studies, 010374 Bucharest, Romania; 4 Education, Training and Labour Market Department, National Scientific, Research Institute for Labour and Social Protection, 010643 Bucharest, Romania

**Keywords:** fine dining, restaurants, consumer behavior, health belief model, sentiment analysis, COVID-19, pandemic, consumer trends, global food trends

## Abstract

This research paper aims to analyse how consumer emotions have evolved during the pandemic period in comparison with the pre-pandemic period in relation to restaurant demand in the Romanian fine-dining industry and uses valuable information based on social-media sentiment analysis and content analysis. Focusing on theories of consumer behaviour, the study aims to emphasize how, under the influence of an epidemic crisis caused by an infectious disease, individual behaviour adapts to the “new normal”, embracing a series of changes in the preferences, attitudes, and cognitive choice-making processes. The article takes into account a comparative analysis of the consumer emotions between the pre-COVID-19 pandemic period (2010–2019) and the pandemic period (2020–present), based on the online reviews provided by customers for five fine-dining restaurants from Bucharest, the capital city of Romania: The Artist, Relais & Chateaux Le Bistrot Francais, Casa di David, Kaiamo, and L’Atelier. The research was based on two mining analyses—content analysis and sentiment analysis—and explored the emotional intent of words, with the data being collected from TripAdvisor through web-scrapping. The empirical results defined the fine-dining experience during the pandemic as being associated with the quality of the dishes and also with the quality of the service. The overall consumer sentiment in the direction of the restaurants analyzed is positive. The sentiment research found that throughout the epidemic, the consumers’ attitudes about restaurants deteriorated. In this sense, consumers seem to be less satisfied with the restaurants’ services than before the pandemic. This is another thing that the restaurants had difficulties in when adapting their operations for the pandemic.

## 1. Introduction

COVID-19 sparked a global public health catastrophe and a series of additional concerns, including an economic downturn, unemployment, and mental instability. The pandemic has affected individuals from all around the world, causing anxiety, stress, worry, dread, repugnance, and poignancy, in addition to the illness [1].

The epidemic has posed an unprecedented challenge to the restaurant industry. Community lockdowns, social distancing, stay-at-home ordering, travel and mobility restrictions, and other strategies to flatten the COVID-19 curve have resulted in the temporary closure of many hospitality businesses and a significant decrease in demand for businesses that were allowed to continue to operate [2].

The concept of “normality” has been completely redefined, leading global economies to the verge of collapse within weeks of the initial occurrence of the virus in Hubei, China in December 2019 [3]. The “new normal”, characterized by fear of illness and uncertainty regarding the foreseeable future, has given birth to a series of new consumer trends and, implicitly, a number of new global trends across different industries affected by the pandemic. The effects of the highly contagious virus could only be diminished by reducing interpersonal contact through social distancing and lockdown, which has caused major disruptions to all industries, especially those that are highly reliant on physical human interaction, including the food service industry.

The global restaurant industry was no exception. Even though the physical distancing rules enforced by local governments, as well as the banning of restaurant dine-in services, have played a key role in alleviating the spread of the SARS-CoV-2 virus, they have also represented a huge threat towards the survival of restaurants worldwide [4] (p. 3810). Almost every restaurant was forced to limit their business exclusively to take-out.

The economic impact of COVID-19 on the restaurant industry has not been properly dispersed. While pizza companies maintained or boosted sales throughout the epidemic, casual-dining and fine-dining restaurants saw revenue drops of up to 85% [5]. For some fine-dining establishments, revenues dropped to zero. This industry is expected to return to its pre-crisis level in Q2 2024, the speed of recovery depending on the success of virus-containment policies and the vaccination rate and being different for various types of restaurants [5].

During a pandemic, several forms of mental worries occur, and sentiment analysis may be used to describe a whole spectrum of emotions. Sentiment analysis is a more effective technique to track public sentiment for active users. Barkur and Vibha [6] look into how Indians felt during the COVID-19 lockdown. They utilized famous hashtags to determine people’s happiness and negativity. Sentiment analysis is a technical study about people’s emotions, opinions, and attitudes [7].

The present research paper has a twofold objective: on the one hand, it aims to provide a comparative exploration of customer sentiments in both the pre-pandemic and the pandemic periods, highlighting which words of emotional and opinion content are important to customers. On the other hand, it aims to analyse how consumer emotions have evolved during the two specified periods of time in relation to restaurant demand in the Romanian fine-dining industry.

In order to do that, the present article takes into account a comparative analysis of consumer sentiments between the pre-COVID-19 period (2010–2019) and the pandemic period (2020–present), based on the online customer reviews for five fine-dining restaurants from Bucharest. Having the “voice of the consumer” at the heart of the study, the primary research combines two mining analyses—content analysis and sentiment analysis—with data being collected from TripAdvisor through web-scrapping. In order to help restaurants maintain and improve their service quality, it is important to analyse the sentiments of the online reviews, which help in providing a better understanding of the opinions conveyed by the diners [8].

Therefore, this paper aims to respond to the following research questions: (RQ1) How do consumers most often describe their fine-dining restaurant experience in the pre-pandemic times and during the pandemic times? (RQ2) What are the most positive and negative words used by the customers in describing the fine-dining experience in both periods? (RQ3) How can the customer sentiment be described in terms of positivity and negativity in both periods? (RQ4) Has there been a change in customer sentiment in the pre-pandemic vs. the pandemic period and is this due to more positivity towards the pre-pandemic or more negativity towards the pandemic?

The focus on fine-dining restaurants relies on the assumption that this specific niche segment of the restaurant industry has, for a number of reasons, been the most affected by the COVID-19 pandemic, especially during lockdown. Firstly, unlike casual restaurants, fine-dining restaurants are not just about the food, but about the entire experience of the in-restaurant dining: the décor of the venue, the setting of the table, the music, the scents, the lighting, the exquisite cocktails, and the show made by the chefs are all defining elements of the luxury dining experience. Customers that visit fine-dining restaurants do not do so only to satisfy their hunger, but to indulge in the fantasy of a narrative that became compromised during lockdown, when all venues were closed to the public. Secondly, plating is a key element in the fine-dining experience. This became an issue during lockdown, because of the chef’s inability to maintain the authenticity of the aesthetic of a restaurant by transferring all the different artistic elements from a plate into a delivery cardboard box.

Although the consumer sentiment has been mostly analysed during the pandemic [9], to the best of our knowledge very few studies have investigated consumer sentiment from online reviews on the fine dining. This study aims to fill this research gap by using text-mining approaches to explore the evolution in consumer sentiments and to analyse the change in the overall sentiment from the fine-dining industry in both periods. Moreover, sentiment analysis is a technical tool used to track brand and product sentiment in consumer feedback and better understand customer demands and has become a crucial tool for monitoring and understanding client sentiment. However, to the best of our knowledge, no empirical research to date has considered a comparative analysis of consumer emotional responses in the Romanian fine-dining industry in the pre-pandemic and during the pandemic. Furthermore, no sentiment polarity has been explored in either of the two periods.

The paper is organised as follows. The section of theoretical development emphasises the most relevant opinions regarding the customer sentiment over time, while the third section is dedicated to the presentation of the data and methodology. The section of empirical results highlights the most relevant findings, and the paper ends with the main implications, limitations, and future directions of the research.

## 2. Theoretical Development

### 2.1. Impact of the COVID-19 Pandemic on Global Restaurant Dining

The pandemic has had a significant impact on all businesses at a global level, with the hospitality industry being one of the most badly hit by the pandemic [10]. Travel restrictions, lockdowns, and stay-at-home orders restricted people’s movement by stringent enforcement, causing major disruptions to the industry [11,12]. It has also been estimated that 13.4 million jobs from the restaurant industry could be affected by the ongoing global pandemic [13].

The food service industry is highly vulnerable during epidemic crises because of its high reliance on physical human interaction [4] (p. 3813). In March 2020, in the early stages of the pandemic, the global table-booking website, OpenTable, launched the State of the Industry website to showcase how COVID-19 has affected restaurants worldwide [14]. The study has been ongoing—the data, which are being continuously collected, are based on a sample of over 20,000 restaurants on the OpenTable network and illustrates the number of seated diners from online, telephone, and walk-in reservations or, in other words, the number of customers that dined in restaurants during the pandemic (2020 and 2021) in comparison with the pre-pandemic days in 2019 [14]. The restaurants provide OpenTable with information on their inventory, which has enabled the company to create a detailed and accurate year-over-year comparison, comparing the same days of the week in 2020 and 2021 “to the same day of the week in 2019 (not the same date)” [15].

The data published by the company clearly indicate a significant drop in restaurant bookings at a global level from mid-March 2020, compared to mid-March 2019: 47% on 15 March 2020, to 83% on 17 March 2020, to a dramatic drop of over 99% in only a few days on 22 March [15]. The percentage of over 99% has been recorded since the beginning of May (3 May 2020) and has not gone below 90% since 20 May [6]. Starting from 21 May, the data show a minor improvement. Between 21 May and 5 June 2020, OpenTable reports a growth of nearly 10% (OpenTable [14]). The percentage improved to below 50% in the 3rd semester of 2020 (49% on 31 July 2020), down to an encouraging 26% on 1 September [14].

Over the last trimester of 2020 and the first trimester of 2021, the booking percentages fluctuate dramatically from as low as 10% on 6 September 2020 to as high as 66% on 1 February 2021 [14]. Looking at the state and country division of the data, it becomes obvious how the pandemic has evolved differently in different corners of the world, depending on the number of infected individuals at different times: for example, on 6 September 2020, when the global restaurant bookings was down by an average of 10% compared to 2019, the percentage in British Columbia was as low as 3%, compared to Hawaii at a complete polar opposite, being as high as 95% [14].

Analysing the changes in the relationships between sustainability and the hospitality industry following the onset of the COVID-19 crisis, Jones and Comfort [10] revealed that that the crisis offered a vision of a more sustainable future with the emphasis being more on environmental and social issues, rather than economic gains, but also on collective, rather than individual, approaches to consumption. However, this vision may pose a major challenge for the industry and for many of its traditional customers.

Foroudi, Tabaghdehi, and Marvi [16] investigated customer perceptions of the shock of the coronavirus pandemic, focusing on the influence that it has had on their emotions, as well as how all these emotions could impact the future desire to visit restaurants. The study revealed that trust is the foundation on which the hospitality industry rests, and that the transformation of the restaurant business needs the enhancement of localization strategies, practices, and performance.

Based on empirical evidence, Gupta and Sahu [17] proved the positive role of innovative training programmes in the hotel industry in India to support guests and employees during the COVID-19 pandemic, boosting consumer confidence and enhancing their intentions to return.

Mason, Narcum, and Mason [18] offer an innovative perspective, from an empirical point of view, on shifts in consumer decision-making behaviors, collecting data for US consumers during pre-pandemic and post-pandemic times and concluding that this sanitary crisis has dramatically altered the consumer needs, shopping behavior, as well as the post-purchase satisfaction level.

There is a public hesitance towards eating out post-COVID-19 and the foodservice providers need to redesign their strategies in order to encourage and attract customers [19].

Consumer behaviour will continue to reshape the restaurant industry. In the case of restaurants, the speedy adoption of new digital ordering systems, delivery, and drive-thru innovations will continue to be vital after the pandemic and will require several changes by restaurant operators. However, despite all the industry efforts, returning to pre-pandemic levels in dining-in trends is unlikely. Restaurant formats will look different after the pandemic. With changing consumer behaviour regarding digital ordering, as well as the drive-thru and delivery cultures, it is not surprising that several restaurant chains have introduced new restaurant formats [20].

The future of the hospitality and tourism industries in the COVID-19 era is currently uncertain; therefore, substantial research is required to evaluate how the industry might recover and survive the “new normal” of the COVID-19 world [21].

### 2.2. Impact of the COVID-19 Pandemic on Local Restaurant Dining in Romania

Social distancing rules have affected economies by reducing the quantity of labour. The most affected work sectors are arts and leisure, hotels, and restaurants, followed by agriculture and business services, activities in which workers rarely use a computer [2]. As previously argued, the food service industry is one of the most susceptible to epidemic disease, because of its high reliance on “human interaction and gatherings” [4] (p. 3813).

Prior to the COVID-19 crisis, the hotel and restaurant industry in Romania was blooming; it had a total of 40,000 entirely Romanian-owned companies, with a total turnover of EUR 5 billion, and an estimated 400,000 employees, representing a total of 10% of the total employees from the private sector (around 190,000 direct employees and an estimated 210,000 employees working in related industries, such as product suppliers, manufacturers, and service providers) [22]. It is crucial to acknowledge not only those directly employed by the restaurant industry but also those working in related industries in order to understand how the crisis caused by the COVID-19 pandemic has not only led to the collapse of restaurants but has also had a crucial impact on a series of other stakeholders in the food service industry, such as the state, the customers, the suppliers, the producers, and the banks [23] (p. 816).

According to statistics released by the Romanian Hotel and Restaurant Association (HORA), Romania was also severely affected by the pandemic. In 2020, the total turnover of the hotel and restaurant industry registered a total decrease of an astonishing 70% compared to the previous year, with over 40% of local businesses being forced to shut down [22]. Out of these, only 10% are anticipated to reopen after the pandemic, with 30% (approximately 10,000 restaurants) remaining permanently closed due to bankruptcy [24].

Forced by the circumstances, the businesses from the food industry sector have adapted their business model in order to survive the new world order [23]. 

### 2.3. Consumer Perceptions in the Context of the COVID-19 Global Shock

Unavoidably, under the influence of a global crisis caused by an infectious disease, the individual’s behaviour will adapt to the new context—the world during and after the pandemic, which will give birth to a series of changes in the preferences, attitudes, and cognitive choice-making processes of a population that is increasingly reliant on online ordering and home isolation [4]. The previous section of the Literature Review focused on the impact, from an industry perspective, that the COVID-19 epidemic crisis has had on restaurant demand. At this point, the emphasis is going to shift towards consumers and the key role that the COVID-19 crisis has played in reshaping their consumer sentiments.

The question is how does the shock of the pandemic influence customer beliefs, and how could those beliefs have an impact on their anticipated emotions—both positive and negative—and affect their future willingness with regard to dining in restaurants [16]? People’s beliefs inform their behavioural intentions [25].

COVID-19 has significantly impacted people’s emotions, meaning that it has had undeniable consequences for individual happiness and achievement (Johnson et al. [26]). In their decision-making processes, people’s choices are often driven by the anticipation of their feelings regarding the upcoming results [27]. Human emotions have been divided into two distinctive categories, located at polar opposites: positive anticipated emotions, referring to an individual’s successful attempt at achieving a goal, and negative anticipated emotions, referring to an individual’s inability to achieve their target [28].

During the pandemic, there has been a decline in positive emotion and a considerable rise in negative emotions, such as anxiety and depression: studies reveal that consumers have become worried about their personal health, as well as the health of their families and loved ones, and concerned about whether they will continue to be able to provide for their basic needs, as well as their loss of freedom [29]. These common concerns have manifested themselves in different ways as consumers, influenced by internal (psychological) and external factors, have gradually adopted new consumption patterns. People are no longer interested in holidays and various other leisure activities, including in-restaurant dining, shifting their entire focus onto protecting themselves and their loved ones from illness [30]. The prolonged rise in negative emotion is not only damaging to societies and economies, but also has a damaging impact on an individual’s immune system [31].

Jim Samuel et al. [32] addressed an issue of public sentiment, which resulted in increased dread and negative emotion, while Yin et al. [33] proposed a framework for analysing the topic and sentiment dynamics caused by COVID-19 from a large number of Twitter postings. A hybrid technique to finding sentiments on ordinary tweets with polarity calculations was developed in a machine-learning-based sentiment analysis [34]. The polarity score was calculated using three sentiment analysers. Using Twitter data, Ahmed et al. [1] proved that both the users’ involvement and their sentiments vary after a particular time.

Tardin [35] evaluated the impact of COVID-19 on the Brazilian food service industry, using topic modelling based on online reviews and identified in the pandemic period four of the most relevant topics describing the customer relationship with restaurants: ‘delivery’, ‘employees’, ‘experience’, and ‘waiter service’. Using the sentiment analysis, the average value of sentiment of the total sample was 1506, highlighting that the overall sentiment of the consumers towards the restaurants is positive. For the pandemic period, the average is lower than the previous periods, and it becomes clear that the sentiment toward the restaurants reduced in the pandemic period. This aspect has been seen as a reflex of the lack of experience of the restaurants with delivery systems or that the restaurants are no longer capable of delivering the same value to the consumer.

The health belief model (HBM) is a theoretical framework used by scholars to “explain and predict health behaviours in public health research” [4]. The model describes how the preventive behaviour of individuals towards illness “can be explained by their risk perceptions and health beliefs” or, in other words, how individuals will act to protect themselves from illness [29] (p. 568). The HBM is positively influenced by three factors—the perceived susceptibility (people will take measures to protect themselves from a disease if they believe themselves to be vulnerable to a specific condition), the perceived severity (people become wary of a disease “if they believe it would have serious consequences” upon their wellbeing), and the perceived benefits (people become cautious and take preventive measures, under the belief that these would reduce the vulnerability or severity of the disease)—and one negative factor—the “perceived barriers or costs” (factors that prevent individuals from taking the health measures that will protect them) [4] (pp. 3812–3813), [29] (p. 568).

The positive factors are visible in the context of consumer behaviour in relation to restaurant dining during the COVID-19 pandemic. Perceived susceptibility captures the individual’s perceived risk of becoming infected with the SARS-CoV-2 virus; perceived severity captures the individual’s perception of the severity of the COVID-19 infection; and the perceived benefits refer to people’s awareness that avoiding restaurant dining will reduce their risk of infection [4] (p. 3813). All of these internal cues to action have also been shaped by external cues, such as risk communication via different mass media channels, health marketing campaigns, and the restrictions enforced by public authorities—full lockdown being one of the most drastic measures taken during the COVID-19 pandemic, aimed at encouraging social-distancing and limiting human interaction and, by doing so, minimising the spread of such a highly contagious virus [4,24].

Ernst and Young have created the “EY Future Consumer Index”, a study based on sentimental analysis of individuals in five key markets that showcases how the COVID-19 pandemic has re-shaped consumer behaviours, creating new consumer segments [26].

The study identifies four major consumer groups that have emerged during the pandemic: save and stockpile (35% of consumers participating in the study): those that are not so much concerned about the present, but worried about the well-being of their families and the long-terms effects that the pandemic will have on their lives; cut deep (27%): those individuals, who have been most affected by the pandemic, leading them to be the most pessimistic about the future and causing them to reduce their financial spending across all categories; stay calm, carry on (26%): people that have not been directly affected by the pandemic and, as a result, have not changed their spending habits; last, but not least, hibernate and spend (11%): consumers that, in spite of being most worried about the pandemic, have been in the best position to deal with it, having the financial power to spend more across all different categories [36].

The E and Y [37] report revealed that consumers worldwide are significantly more concerned than they were before the pandemic, arguing that we are witnessing the birth of the “Anxious Consumer.” When looking at Chinese consumers, the study pointed out that they were able to return to their “normal life”, but in order to facilitate the adaption to this new reality, companies needed to significantly accelerate digital investment in operations and experiences that helped make the consumers feel safe.

Based on Yelp online reviews from January–June 2020, Luo and Xu [8] found out that customers elicited a higher level of positive feeling for service in March compared to the previous two months; a potential reason might be that the customers tended to take a restaurant’s precarious position into account before evaluating the service quality of a restaurant.

Despite the fact that restaurants reopened in May with capacity limitations and social-distancing guidelines, patrons were found to have a generally good view of their eating experiences. In June, sentiments regarding food and place hit an all-time high, indicating that the restaurants were doing an excellent job of maintaining consistency in their performance criteria. One potential recommendation to boost the customers’ good feelings about ‘location’ might be if the restaurateurs provided ample parking to accommodate the customers. Furthermore, having outside seating may make it easier for consumers to locate the restaurant and eat outside in the fresh air.

Although the pandemic has had the greatest impact on full-service restaurants, altering societal attitudes and commercial environments will permanently transform all the different restaurant categories. Rather than the establishment of a new business model, the coronavirus may have the most dramatic influence on existing operators’ services.

Hospitality businesses are expected to make substantial changes to the way they operate in the COVID-19 business environment in order to ensure employee and customer health and safety and enhance the customers’ willingness to patronize their businesses [38].

The phantom kitchens would be the food-truck counterpart of COVID-19 (i.e., a new sector pushed by a catastrophe). Hundreds of ghost kitchens have emerged in the last year, either as part of established restaurant chains or as stand-alone businesses, estimated to reach an almost USD 1 trillion commercial potential by 2030 [39].

Operators from the limited, as well as the full-service, spectrum are looking forward to a return to dine-in business. Even models that place a large focus on in-person encounters claim that customer demand for off-premises services will persist long beyond COVID-19.

In the restaurant sector, different types of restaurants will soon have less to do with operations and more to do with establishing a specific feeling and brand identity. It has now become mandatory to break down what dining really is, paying particular attention to a guest’s needs and their willingness to have whatever their heart desires, whenever they desire it.

Many of the safety precautions implemented as a result of the pandemic will continue whenever businesses resume, and the fine-dining sector will maintain, and potentially extend, its focus on outdoor eating [39].

Fine-dining establishments that are still closed, have limited visitor counts, or do not provide delivery/to-go options are under the most strain. Many of these concepts are still seeing 30% to 40% same-store sales drops and will likely only see a modest rebound over the next few months as macro forces begin to replace COVID-19 worries, and customers continue to be wary of dine-in experiences [20].

According to the preliminary findings of longitudinal research undertaken by the editorial team of the *Journal of Hospitality Marketing & Management*, a big percentage of people (more than 50%) are unwilling to eat in a restaurant right away [40].

The pandemic is far from over, and if the previous year has taught us anything, it is that no amount of preparation can prevent what is yet to come. It is too early to predict how the foodservice industry will develop, how restaurants will define themselves and differentiate themselves from competitors, and what consumer expectations will be. Whatever the case may be, the sector will rebound with a diverse range of cuisines, styles, service methods, and hospitality. The power to adapt is what will maintain the sector’s long-term survival. From an industry perspective, restaurants, which are highly reliant on face-to-face human interaction, have been forced to reconsider their marketing strategies and adapt to the “new normal”, engaging their customers in virtual communities through social media platforms [41].

KAIAMO, known as the ultimate fine-dining culinary experience, renowned for luxury and sophistication, as well as the artistic plating and exquisite taste, was one of the first restaurants in Bucharest to respond to the changes in consumer behaviour and adapt their business model to the new context, shifting from luxurious in-restaurant dining to dishes that are suitable for take-away and delivery. Soon after the instauration of lockdown in Romania, the restaurant used their Instagram page to announce that the brand is now “Reborn. Refined. Redefined” [42]. Soon after the announcement, the new aesthetic of the brand became visible on Instagram. KAIAMO launched Kaiamo Kanteen, which is, as the word “canteen” suggestively indicates, a less artistic, more realistic version of the original pre-pandemic restaurant, serving dishes from a new menu that focuses less on presentation and more on serving bigger portions at more affordable prices. In other words, there has been a notable shift in their strategy, from unaffordable luxury to “deliverable” dishes, from artistic presentation to comfort food, which is much needed during difficult times. The new culinary aesthetic, as well as the focus on the virtual engagement of customers through online communities, has been visible on the Kaiamo Kanteen Instagram page from the outset of the pandemic: the photos clearly showcase comfort food, instead of artistic dishes, and customer reviews are posted to emphasize the restaurant’s ability to empathize with the people during challenging times. Moreover, the restaurant also partnered with the delivery companies Tazz by Emag, Food Panda, and Glovo to offer home-delivery services, which is something that the “pre-pandemic” KAIAMO did not provide to their clients [42]. 

The Artist, another renowned fine-dining restaurant analysed in the present paper, is another example of a business that has adapted to the new customer behaviour. As a result of the lockdown, the restaurant launched a new menu, specifically designed for home delivery. Moreover, a special home-delivery Christmas menu has also been designed, aiming to bring the luxury experience specific to their restaurant into the safe environment of their customers’ homes [43]. The concept was launched under the name of “The Artist @ Home” and, just as in the case of KAIAMO, it has been promoted on the restaurant’s Instagram page, once again proving that businesses have understood the new customer behaviour and adapted their “pre-pandemic” business model to serve the “pandemic” needs [43].

KAIAMO and The Artist are proof that luxury restaurants have found ways to manage the crisis caused by the pandemic by adopting their business models to the new rules and regulations, as well as to the more hesitant and worried behaviour of their customers. [8,44] Given that the dark cloud of the pandemic is still ongoing, the recommendation based on the findings of this research is that all fine-dining restaurants should embrace adequate mechanisms in order to survive in the foreseeable future—adaptation of menus for home-delivery and engagement of customers through online communities being two of the recommended survival tools.

## 3. Materials and Methods

In order to explore how the customer sentiment evolved over the period, the analysis was based on the online reviews collected for 5 fine-dining restaurants from Bucharest, using the following criteria: the top 100 Restaurants in Bucharest and those ranked $$$$ on TripAdvisor, namely The Artist, Relais & Chateaux Le Bistrot Francais, Casa di David, Kaiamo, and L’Atelier. The sample of reviews extracted from TripAdvisor through web-scrapping is formed from a total of 1106 reviews, the earliest review being from 19 May 2010, while the latest dates from 8 March 2021. The timeframes are considered to be the periods from 2010–2019 (for pre-COVID-19 pandemic) and 2020–the present (during the pandemic). The research will take into account a comparative analysis of consumer sentiment for these two periods. 

**Figure A1:**
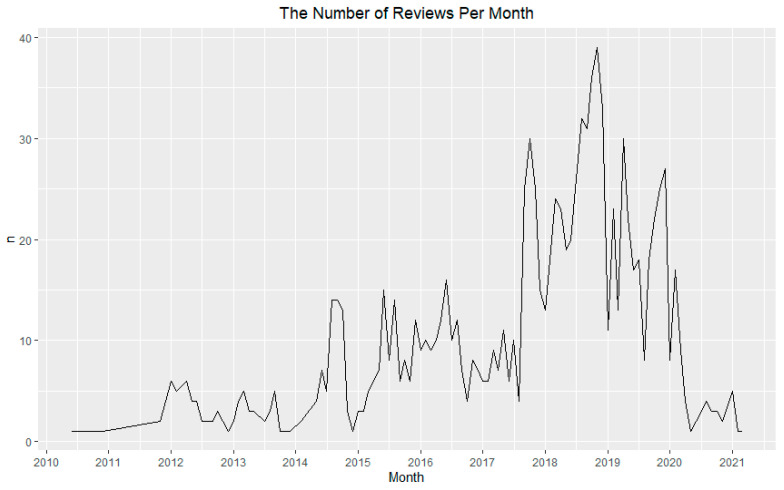
Total number of reviews per month.

The sample of reviews extracted from TripAdvisor through web-scrapping consists of a total of 1106 reviews for five restaurants, the earliest review being from 19 May 2010, while the latest dates from 8 March 2021. The evolution of the total number of reviews per month highlighted a continuous increase in the number of reviews from 2010 until the end of 2018, with, at the end of 2018, a sharp decline for the last period (Figure A1 from Appendix A).

The distribution of reviews per restaurant revealed that Casa di David is the oldest luxury restaurant while Kaiamo is the newest restaurant, with reviews starting from 2018 (Table A1 from Appendix A). Analysing the frequency of reviews per restaurant, we can mention that most of the reviews for the whole period were acquired by “The Artist”, with almost 400 reviews, followed by “Relais & Chateaux Le Bistrot Francais” and “Casa di David”; at the opposite side we encountered “Kaiamo”, a relatively new restaurant.

Comparing the consistency of reviews before the pandemic (until 2019) and during the pandemic (2020–March 2021), the research reveals a sharp decline in client reviews as a result of the strict measures imposed by the pandemic—measures that also included closing down restaurants.

When customers leave feedback—whether it is to complain or leave a flattering review—there is always an underlying emotion. Having access to the right data at the right time can be a game-changer when it comes to making decisions that boost customer satisfaction and loyalty.

Online reviews are still as crucial as ever during COVID-19. During the COVID-19 crisis, timely online reviews might assist potential customers in getting the most up-to-date information about how a restaurant is running. A single poor review might discourage potential customers, making it much more difficult for a food business to survive the COVID-19 crisis.

Online reviews have become one of today’s most powerful marketing tools, influencing customer behaviour with an astonishing 91% of 18–34-year-old consumers claiming that they trust online reviews as much as personal recommendations [45,46]. Consumer online reviews play a critical role (Del Chiappa et al. [47]; Liu and Park [48]) and have important consequences from a managerial point of view. Customers become “objective voices” (Vermeulen and Seegers [49]), with more than 75% of consumers taking peer reviews into account when planning a holiday (Xie et al. [50]; D’Acunto, Tuan and Dalli [51]). A study by D’Acunto et al. [51], based on a sample of TripAdvisor reviews, analyzed the usefulness of the reviews.

From the customers’ perspective, online reviews empower them to express their opinion, providing social proof to other potential clients [45]. From an industry perspective, the customers’ online reviews can have either a positive or a negative impact on their business. On the one hand, positive reviews have the power to increase customer trust and improve customer experience, whilst on the other hand, negative reviews have the power to reduce the customer base, with 94% of consumers agreeing that a negative review has convinced them to avoid using a specific business [45].

Published literature clearly reveals a division when it comes to the reliability of online customer reviews. On the one hand, the role of customer reviews as rich material is acknowledged, complementing or substituting for existing information sources, whilst on the other hand, they can express the subjective opinion of a limited sample of customers [45].

Online reviews are an effective word-of-mouth marketing strategy in the digital age, providing outside perspectives on products and services. While positive reviews can drive revenue and build a trustworthy reputation, negative reviews, or the absence of reviews, can do the opposite. Understanding the importance of reviews, as well as how to leverage them to boost the business can be a critical way to get ahead in the competitive e-commerce marketplace and be positioned miles ahead of the competition. Therefore, even if there are pros and cons, we decided to use the online reviews from TripAdvisor as the basis for this research paper. A study conducted in partnership with Ipsos MORI polled over 23,000 TripAdvisor users from 12 markets, “across hotel, restaurant and attraction reviews, revealing that more than four out of five (85%) participants report that the reviews they read on TripAdvisor accurately reflect their experience, and 86% agree that TripAdvisor makes them feel more confident in their booking decisions [52]. Moreover, the results of Chua and Banerjee (2013) supported the same conclusion [53].

The research methodology combines elements from two relevant mining analyses—content and sentiment analyses—in order to explore customer sentiment in both the pre-pandemic and the pandemic periods, highlighting which words of emotional and opinion content are important to customers, analysing how consumer emotions evolved in these two periods in relation to restaurant demand in the Romanian fine-dining industry.

On the one hand, content analysis is a technique that extracts worthwhile information and represents a solution for the unstructured data. It represents a method of systematic research designed to analyse and infer text, exploring the meanings of different words, themes, or concepts based on word-cloud analysis. Word clouds are visualizations that display words and word frequency to gain an understanding of what consumers specifically like or dislike about a location.

On the other hand, the sentiment analysis revealed the emotional tone behind the words used to understand the attitudes, opinions, and emotions and extracted insights from the social data.

When human readers approach a text, the understanding of the emotional intent of words is used to infer whether a section of text is positive or negative. The text is considered as a combination of individual words and the sentiment content of the whole text as the sum of the sentiment content of the individual words. Certain emotions are strongly related to specific words. In the sentiment analysis, the proportions of the words that have positive connotations or negative connotations, or are neutral, present interest, and an exploration is made of how many words in a text are also in a predefined list of words associated with a certain sentiment. Within the paper, we have applied sentiment analysis using unsupervised learning, in which the content is characterized by given words or dictionaries; we used the AFINN lexicon dictionary in R software, developed by Finn Årup Nielsen, for a list of words which consists of 2477 coded words and word scores ranging from −5 (very negative) to +5 (very positive). As preliminary steps, AFINN preprocessed the text by removing the punctuation and converted all the words to lower case before analyzing it. One of the drawbacks of using the raw AFINN score is that the longer texts may yield higher values simply because they contain more words. To avoid that issue, we divided the score by the number of words in the text.

In order to explore how customer sentiments evolved during the pandemic and to explore potential changes over time, the first step implied the computation of an average sentiment score for both periods, pre-pandemic and pandemic, using the formula:

(1) $$\mathrm{Average}\;\mathrm{sentiment}\;\mathrm{score}=\frac{\mathrm{Sum}\left(\mathrm{positive}\right)-\mathrm{Sum}\left(\mathrm{negative}\right)}{\mathrm{Total}\;\mathrm{Words}\;\mathrm{Count}}$$

Each review was given a sentiment score based on how positive or negative the review was. The final sentiment score ranged between −5 to 5, with the assigning of the following sentiment categories: neutral for a score ranging from −1 to 1, positive for a score ranging from 1 to 2.5, very positive for 2.5 to 5, negative for a score ranging from −1 to −2.5, and very negative from −2.5 to −5. In the second step, based on the values of the average sentiment score, we applied the Welch two-sample *t*-test in order to highlight reliable change in the average sentiment in both periods, as well as a two-sample, difference-of-proportions *t*-test to highlight significant differences in terms of positivity and negativity between both periods.

The sentiment analysis can experience some difficulty in understanding a few intricacies of human language—polarity, sarcasm, emojis, comparative sentences, or double negatives—referring to many in-between terms, such as “not so bad” or “kind of good”, which imply average emotion (mid-polarity), and usually, the sentiment analysis fails to pick up on these emotions. Moreover, sentiment analysis is not able to detect any sarcasm in the comments, it being difficult for the tool to detect the real context behind the sentence, or a double negative, which turns the sentence into a positive.

All analyses were developed using libraries tm, tidyte xt, quanteda, tidyverse, corpus, textmineR, tidyr, Rweka, wordcloud2, igraph, ggraph, widyr, stats, ldatuning, stm, readr, readtext, reshape2, ape, and dendextend in R statistical software [54].

## 4. Empirical Results

### 4.1. Empirical Results of Content Analysis

Customers frequently leave reviews to describe their experiences, which can be key indicators of what specific problems a business is having. Being able to comprehend thousands of reviews through customer sentiment analysis can help identify patterns and behaviors to help improve a restaurant’s performance.

**Figure 1:**
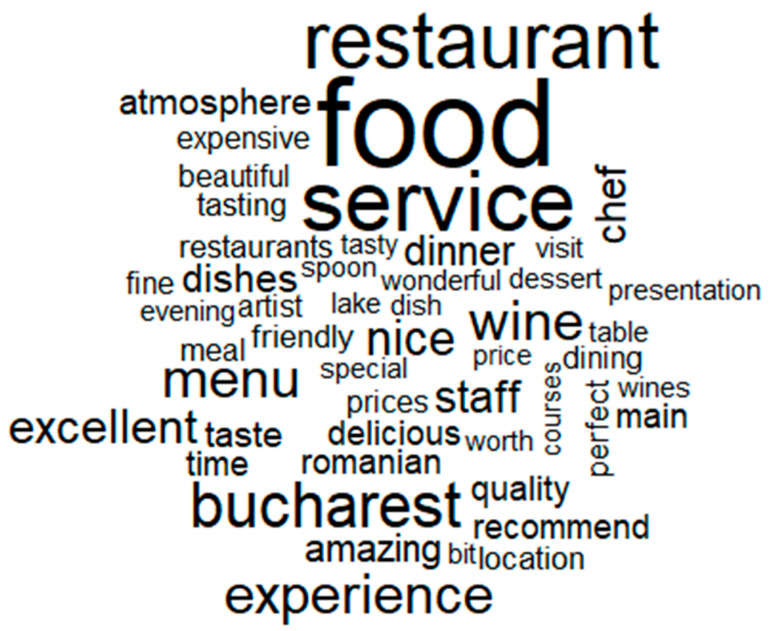
Word cloud analysis for the whole period for all five restaurants.

In order to identify the ways in which customers describe their fine-dining restaurant experience in general as well as in the pre-pandemic and during the pandemic (RQ1), the word-cloud analysis revealed that the most common words characterizing all fine-dining restaurants from our sample are: “food”, “restaurant”, “service”, “Bucharest”, “experience”, “menu”, “wine”, and “excellent” (Figure 1).

**Figure 2:**
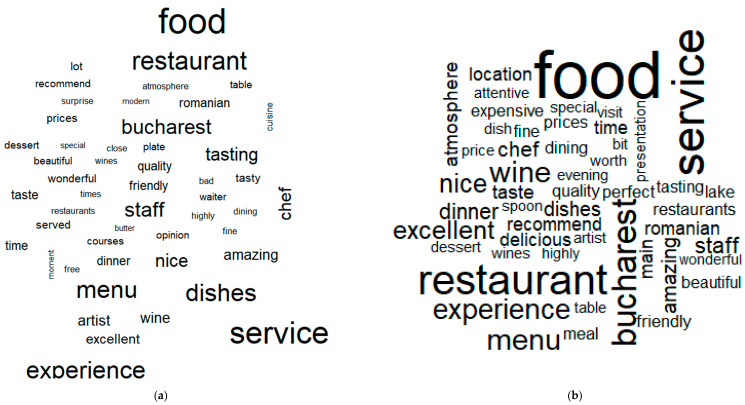
Word cloud analysis for all restaurants before and during the pandemic: (**a**) before the pandemic; (**b**) during the pandemic.

Analysing the distribution of the most positive and negative words before and during the pandemic, the following can be highlighted (Figure 2):

- the words characterizing the fine-dining restaurants pre-pandemic have been “food”, “service”, “restaurant”, “Bucharest”, “experience”, “menu”, and “wine”;
- during the pandemic, it can be observed that even if the restaurants registered a sharp decline in the total number of reviews because of the restrictions and lockdown, the words characterizing the fine-dining restaurants remain almost the same, namely “food”, “restaurant”, “service”, “menu”, “dishes”, “experience”, “Bucharest”, “tasting”, “staff”, or “chef” Therefore, the fine-dining experience in a pandemic is more likely to be associated with the quality of the dishes and also with the quality of service.

### 4.2. The Empirical Results of Sentiment Analysis

**Figure 3:**
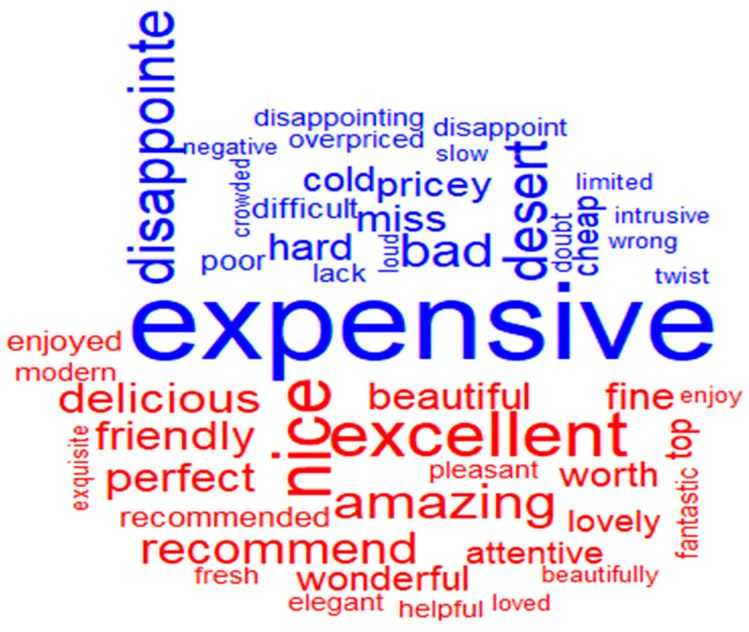
Most common positive and negative words in the total number of reviews for the whole period of time.

Sentiment analysis provides a way to understand the attitudes and opinions expressed in texts. Analysing the whole sample of reviews, it is possible to highlight that the most common negative word is “expensive”, followed by “disappointed”, “dessert”, “bad”, “pricey”, and “cold” (Figure 3).

At the opposite pole, there are the most common positive words for the entire sample, namely “excellent”, “nice”, “amazing”, “recommend”, “delicious”, “friendly”, “perfect”, “beautiful”, “wonderful”, or “fine” (Figure 3).

**Figure 4:**
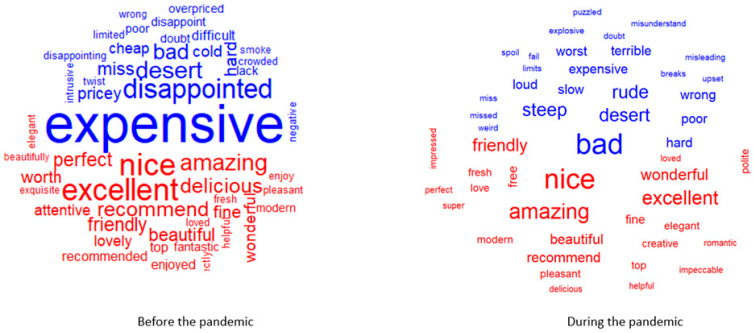
Most common positive and negative words in reviews before and during pandemic.

In order to respond to RQ2, a comparative analysis of the word clouds before and during the pandemic in terms of the most common positive and negative words reveals that (Figure 4):

- if before the pandemic, the most common negative word was “expensive”, followed by “disappointed”, “dessert”, “bad”; during the pandemic these were replaced by “bad”, accompanied by “dessert”, “rude”, and “steep”;
- in terms of the most common positive words, before the pandemic these were “excellent”, “nice”, “amazing”, and they remained the same during the pandemic (“nice”, “amazing”, “wonderful”, “excellent”).

**Figure 5:**
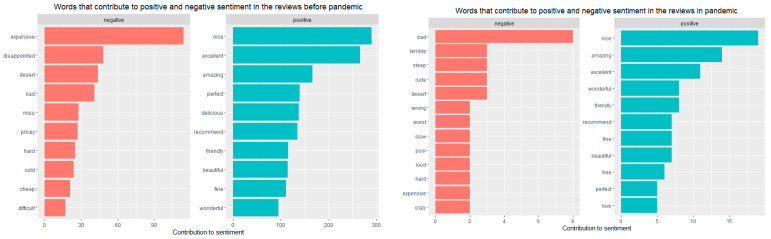
Words that contribute to positive and negative sentiment in the reviews before and during the pandemic.

It is also worth investigating how customer emotions evolved in terms of the most common words contributing to the positive and negative sentiments in the pre-pandemic period vs. the pandemic period (Figure 5) in order to respond to RQ3.

By doing so, one can clearly observe that prior to the pandemic the most common positive words were “nice”, “excellent”, and “amazing” and that they preserve their meaning even during the pandemic. If before the pandemic we identified words such as “expensive”, “disappointed”, “dessert” as the most commonly used negative words, during the pandemic we found words such as “bad”, “terrible”, and “steep”.

From the positive word clouds, it can be observed that the fine-dining experience continues to be defined by the same attributes, with the customers acknowledging that the experience is nice, excellent, and amazing, as those are the largest words in the cloud. However, knowledge gained from the negative word cloud may be more insightful. Before the pandemic it can be observed that some of the larger words include “expensive”, “disappointed” and “dessert”. If people are using the word “dessert” in their reviews, it may indicate that many customers experienced some issues with the dessert, being disappointed by the quality or finding it too expensive. During the pandemic one can observe an accentuation of the negative feeling, emphasized through the usage of words such as “bad”, “terrible”, “steep”, also revealing issues related to prices.

**Figure 6:**
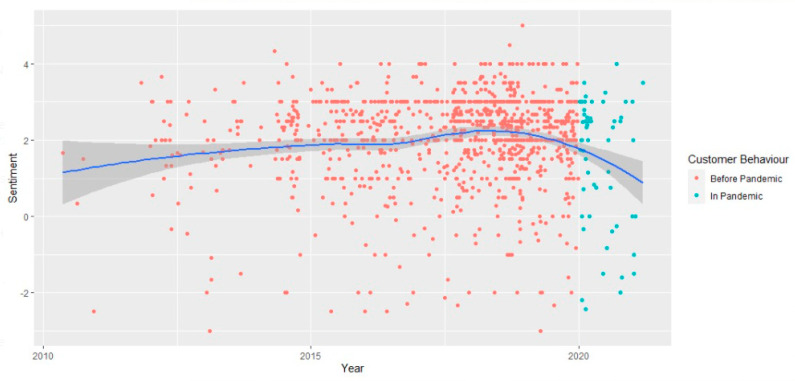
The customer sentiment score over time.

**Figure A2:**
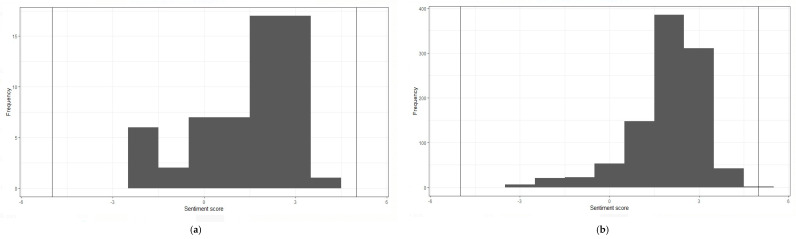
Histogram of sentiment score before and during pandemic: (**a**) before the pandemic, (**b**) during the pandemic.

Furthermore, in order to respond to RQ4, a customer sentiment score was built based on how positive or negative the reviews were. In order to explore whether the sentiments of the customers evolved over time, the distribution of the average sentiment score for both periods, as well as the evolution of the sentiment score over time, together with the empirical results of the Welch two-sample *t*-test were explored. Thus, the modal sentiment (the most frequent sentiment), even if characterized by a small decrease, was slightly positive from an average of 2.03 in the pre-pandemic to almost 1.49 during the pandemic, revealing a decrease in the degree of positivity (Figure A2 from Appendix A). An analysis of the evolution of the customers’ sentiments pre-pandemic, as well as during the pandemic, reveals the same decrease in the positivity (Figure 6).

The results are in line with the conclusions of Tardin [35], highlighting that the overall sentiment of the consumers towards the restaurants is positive and that the sentiment towards the restaurants reduced in the pandemic period, especially with regard to the lack of experience of the restaurants with delivery systems or to the restaurants not being capable of delivering the same value to the consumer.

The empirical results of the Welch two-sample *t*-test (Table 1) reinforced the same conclusion of a true statistical difference in the average customer sentiment across both periods as the probability of the Welch *t*-test was smaller than the 5% significance level.

Whether this difference is due to more positivity towards the pre-pandemic or more negativity towards the pandemic can be determined using the two-sample difference-of- proportions *t*-tests. (Table 2).

The empirical results of the *t*-tests for both the differences in positivity and in negativity revealed that statistically significant differences can be observed for both the positivity and the negativity degree, the probabilities (*p*-values) being smaller than a 1% significant level. Additionally, the difference in the ratios is for positivity 0.157, meaning that the pre-pandemic period has a 0.157 higher ratio of positive sentiments compared to the pandemic period. For the negativity degree, the pre-pandemic negative ratio is 0.087 higher than for the pandemic; this difference is also statistically highly significant.

Therefore, we can conclude that the level of positivity slightly decreased during the pandemic, and so did the level of negativity. A potential explanation for this finding can rely on the fact that positive and negative emotions occur concurrently in a consumption experience [55] as the occurrence of blended emotional experiences has been demonstrated in the consuming of food [56]. Cacioppo et al. [57], Penz and Hogg [58], and Pang et al. [59] have recently proposed that humans can experience various emotions at the same time but of opposing valence. Customers react to extravagant consumption with a combination of emotions, both good and negative, according to Ramanatham and Williams [60]. Manthiou, Hickman, and Klaus [55] (p. 102218) explained very well the co-occurrence of positive and negative emotions, stating that “a couple has dinner at a fine-dining restaurant; although the atmosphere and decoration are splendid, they have to wait long for their main course. The wine is excellent, but the meat is overdone. The salad ingredients are fresh, but the salad dressing is inadequate. The dessert is delicious and delivered on time, but the two servers are not equally reliable and helpful. Will these customers depart feeling satisfied, dissatisfied, or both”?

The ambiance was the attribute with the largest improvement in guest sentiment, in conjunction with the change to the growth of off-premises sales as dine-in activity fell alongside the increase in COVID-19 cases. Traditionally, off-premises food sentiment has always been lower than dine-in. All these could be reasons for the decrease in the positivity between both periods. During the pandemic, safety remains the customers’ primary concern and meeting or exceeding expectations for safety can contribute to positive feedback online.

## 5. Conclusions and Implications

COVID-19 has been the restaurant industry’s greatest challenge to date, as well as a severe public-health issue. Never before have such a large number of restaurants been forced to close, some of which will never reopen. Consumer demand will most certainly not grow instantly when restrictions are eased, according to early signs from China and other nations where the epidemic appears to be under control. Restaurants that prepare ahead and are ready to adapt and develop their business model in accordance with the “new normal” will be in a better position to restore pre-crisis sales levels. The study examined online reviews on Romanian fine-dining restaurants, discovering the underlying aspects related to the consumer experiences in these restaurants through content analysis and examining how the customer sentiment evolved over time, based on the results of sentiment analysis.

The crisis caused by the COVID-19 outbreak is still an ongoing issue and the foreseeable future is uncertain. Whilst the data clearly indicate that the global economy is entering a recovery stage, scholars and industry experts claim that at the moment the “global trends” and “consumer trends” are nothing but predictions as no one can know for sure how industries, as well as consumers, will re-adapt to “normality” after witnessing the huge pandemic shock.

As expected, during the pandemic there has been a sharp decline in client reviews, as a result of the strict measures imposed by the pandemic—measures that also included closing down restaurants. However, Casa di David has been the most constant in receiving reviews, while The Artist has been the restaurant with the highest number of reviews.

In describing the customers’ fine-dining restaurant experience, it can be mentioned that this experience is mostly related to words such as “food”, “restaurant”, “service”, “Bucharest”, “experience”, “menu”, “wine”, and “excellent”. If we explore this experience in a comparative way, before and during the pandemic, it can be highlighted that the fine-dining experience before the pandemic is characterised by “food”, “service”, “restaurant”, “Bucharest”, “experience”, “menu”, and “wine”, while in the pandemic the words characterizing the fine-dining restaurants remain almost the same, namely “food”, “restaurant”, “service”, “menu”, “dishes”, “experience”, “Bucharest”, “tasting”, “staff”, or “chef”. Therefore, the fine-dining experience in a pandemic is more likely to be associated with the quality of the dishes as well as with the quality of service.

In terms of negative sentiments, the fine-dining experience could be characterised before the pandemic by “expensive”, “disappointed”, “dessert”, “bad”, while during the pandemic these have been replaced by “bad”, accompanied by “dessert”, “rude”, and “steep”.

In terms of positivity, the most common positive words in the pre-pandemic were “excellent”, “nice”, and “amazing”, and they remained almost the same during the pandemic (“nice”, “amazing”, “wonderful”, and “excellent”).

The overall consumer sentiment in the direction of the restaurants analyzed is positive. The COVID-19 pandemic severely affected the Romanian restaurant business, as shown by the radical decrease in the number of reviews.

Even while the restaurants continued to operate through delivery systems, it appears that this was insufficient to maintain their customers. The sentiment research found that throughout the epidemic the consumers’ attitudes about restaurants deteriorated. In this sense, the consumers seem to be less satisfied with the restaurant’s services than before the pandemic. This is another thing that the restaurants had difficulties in when adapting their operations for the pandemic (Tardin [35]).

Managers in the food service business will benefit from the findings of this study.

The results of the content and sentiment analysis clearly highlighted the most relevant aspects that consumers express interest in with regard to restaurants, showing paths for managers to facilitate and improve their restaurant’s operations. The most common element mentioned by the customers is related to the price, which is associated with the lowest associated customer sentiment. Nonetheless, the COVID-19 context changed the socioeconomic environment in which restaurants operate. Consumers are evaluating the delivery aspect, and managers should keep up with this new demand.

This research is one of the few types of research utilizing text mining together with sentiment analysis to examine consumer sentiments about the Romanian fine-dining industry, especially with regard to understanding the impacts of the COVID-19 pandemic on this sector.

Despite this paper’s contributions, one key question remains open: is there going to be a fundamental change in customer behavior towards the food service business after the epidemic ends and everything returns to ‘normal’?

## 6. Limitations and Future Directions of Research

A first limitation of this research is the small number of reviews of the pandemic period. Collecting more data from this period can significantly change the results. The review website selection is also a limitation of this paper. The selection of only TripAdvisor could induce a platform bias. Therefore, future research can investigate the impact of COVID-19 on other platforms as well. Another limitation was the number of restaurants chosen.

Last, but not least, the present article focused on discussing consumer sentiment changes during the pandemic (2020–present), in comparison to the pre-pandemic period (2010–2019). However, with the world economy now entering a recovery stage, consumer behaviour will be subject to periodic change, depending on the progress of the pandemic as well as the ability of individuals to adapt to the ever-changing “new normality”. As a result, year-by-year analysis is recommended for the next five years in order to gain a deeper understanding of what the post-pandemic consumer looks like.

Future directions of research include developing the analysis further to include restaurants of different price points, located in different cities in Romania, in order to gain a deeper contextual understanding of the current consumer sentiment in relation to the restaurant industry at a national level.

## Figures and Tables

**Table 1:** The empirical results of Welch two-sample *t*-test.

t = 2.384.	df = 59.41	*p*-value = 0.02
alternative hypothesis: true difference in means is not equal to 0		
95 percent confidence interval: 0.08 0.98		
mean of x = 2.03 mean of y = 1.49		

**Table 2:** The empirical results of two-sample difference-of-proportions *t*-test.

**Positivity**		
t = 7.78	df = 1	*p*-value = 0.00527
alternative hypothesis: true difference in positivity proportions is not equal to 0		
95 percent confidence interval: 0.0229 0.2908		
proportion of positivity in pre-pandemic = 0.823 proportion of positivity in pandemic = 0.666		
**Negativity**		
t = 10.62	df = 1	*p*-value = 0.0011
alternative hypothesis: true difference in negativity proportions is not equal to 0		
95 percent confidence interval: 0.0013 0.173		
proportion of negativity in pre-pandemic = 0.964 proportion of negativity in pandemic = 0.877		

## Data Availability

The main source for the data supporting the reported results can be found on the TripAdvisor website for each restaurant considered within the analysis.

## Appendix A

**Table A1:** The distribution of reviews per restaurant.

Restaurant	First Review	Latest Review
Casa di David	19/05/2010	17/01/2021
Kaiamo	14/09/2018	29/12/2020
L’Atelier	27/07/2014	08/03/2021
Relais & Chateaux Le Bistrot Francais	24/05/2014	17/10/2020
The Artist	25/08/2017	11/10/2020
